# Mapping the FACT-G cancer-specific quality of life instrument to the EQ-5D and SF-6D

**DOI:** 10.1186/1477-7525-11-203

**Published:** 2013-12-01

**Authors:** Paulos Teckle, Helen McTaggart-Cowan, Kim Van der Hoek, Stephen Chia, Barb Melosky, Karen Gelmon, Stuart Peacock

**Affiliations:** 1 Canadian Centre for Applied Research in Cancer Control, British Columbia Cancer Agency, Vancouver, BC, Canada; 2 Cancer Control Research, British Columbia Cancer Agency, Vancouver, BC, Canada; 3 School of Population and Public Health, University of British Columbia, Vancouver, BC, Canada; 4 Medical Oncology, British Columbia Cancer Agency, Vancouver, BC, Canada

**Keywords:** Cancer-specific instrument, Cross-walking, Mapping, Quality adjusted life-years, Regression modeling, Utility assessment

## Abstract

**Objective:**

To help facilitate economic evaluations of oncology treatments, we mapped responses on cancer-specific instrument to generic preference-based measures.

**Methods:**

Cancer patients (n = 367) completed one cancer-specific instrument, the FACT-G, and two preference-based measures, the EQ-5D and SF-6D. Responses were randomly divided to form development (n = 184) and cross-validation (n = 183) samples. Relationships between the instruments were estimated using ordinary least squares (OLS), generalized linear models (GLM), and censored least absolute deviations (CLAD) regression approaches. The performance of each model was assessed in terms of how well the responses to the cancer-specific instrument predicted EQ-5D and SF-6D utilities using mean absolute error (MAE) and root mean squared error (RMSE).

**Results:**

Physical, functional, and emotional well-being domain scores of the FACT-G best explained the EQ-5D and SF-6D. In terms of accuracy of prediction as measured in RMSE, the CLAD model performed best for the EQ-5D (RMSE = 0.095) whereas the GLM model performed best for the SF-6D (RMSE = 0.061). The GLM predicted SF-6D scores matched the observed values more closely than the CLAD and OLS.

**Conclusion:**

Our results demonstrate that the estimation of both EQ-5D and SF-6D utility indices using the FACT-G responses can be achieved. The CLAD model for the EQ-5D and the GLM model for the SF-6D are recommended. Thus, it is possible to estimate quality-adjusted life years for economic evaluation from studies where only cancer-specific instrument have been administered.

## Introduction

Cancer is now the leading cause of death in many developed countries including Canada [1]. It accounted for 30 per cent of all deaths in 2008, followed by heart disease (21%) and stroke (6%) [2]. Over the past 50 years, survival rates for many forms of cancer have improved markedly due to advances in surgery, radiation therapy, and chemotherapy [1]. However, emerging cancer therapies often come at high costs. This not only imposes an increasing financial burden on health care systems but also raises questions about how to assess best value for money [3,4]. Economic evaluation is used by decision-makers in Canada and other countries to inform the allocation of scarce resources across health care interventions. Specifically, Cost-Utility Analysis (CUA) is increasingly becoming the main approach used to measure and value the impacts of interventions. CUA uses quality-adjusted life years (QALYs) to measure health outcomes by combining survival and health-related quality of life (HRQoL) into a single index. The use of CUA is recommended by the Canadian Agency for Drugs and Technologies in Health (CADTH), as well as peak regulatory bodies in Australia, the UK, and elsewhere in Europe [5-10].

The Functional Assessment of Cancer Therapy-General (FACT-G) is one of the most widely used cancer-specific HRQoL instrument [11]. It has been validated across a wide range of different types of cancer patients, cultures, and languages, and can be used to assess the impacts of cancer and its treatment on the physical and psycho-social well-being of patients [11]. In spite of its widespread use in clinical trials, responses from the FACT-G cannot be readily used in economic evaluations because it does not provide a single preference-based index of HRQoL suitable for use in CUA. Health utilities, elicited using, for example, the EuroQol-5D (EQ-5D) and the Short Form-6D (SF-6D), provide preference weights from the general population that can be used to calculate QALYs for CUA; this can inform decisions of health-related resource allocation [12,13]. However preference-based instruments typically cover broader health dimensions, and are not often administered in cancer clinical trials because many of the dimensions may neither be relevant nor sensitive to treatment effects [14].

When preference-based instruments are not administered in clinical trials, comparing effectiveness across different interventions becomes cumbersome. One option to overcome this limitation is to map, or ‘cross-walk’, the responses from a disease-specific instrument into a preference-based measure using regression modeling [15,16]. In recent years, there has been growing interest in mapping in the literature, with a number of publications that have mapped responses on disease-specific instruments to generic preference-based measures [17-26]; these studies are further described in two recent review articles [15-27]. In the area of oncology, we have identified 13 studies that have mapped responses from cancer-specific instruments to yield utilities [28].

Our review revealed that only three studies mapped FACT-G responses to preference-based instruments. Two of these studies used responses from patients with a single tumor type (e.g., colorectal [29], prostate [30]; whereas, the remaining study performed the mapping exercise using a general cancer population with the EQ-5D as the target measure [22]. Given that the HRQoL of patients with different types of cancer (and different stages of disease) can vary considerably, it is possible that the results of mapping exercises may differ depending on the type of cancer patients included in the study. As such, further investigation is needed to explore whether a more appropriate mapping function can be developed using responses from patients with different tumor types compared to a specific population. The resulting algorithm may better facilitate future CUAs because it may be more applicable in general cancer populations.

Thus, the objective of this study is to develop a mapping function to impute both EQ-5D and SF-6D health utility values from responses on the FACT-G using a sample of patients with cancer from three different sites (breast, colorectal, and lung) with a range of disease severity. The influence of the tumor sites, as well as disease severity, was investigated.

## Methods

### Study population

To participate in the study, patients had to meet the following criteria: be diagnosed with either breast, colorectal, or lung cancer; be 18 years and older; be able to speak and read English; have a life expectancy of at least six months; be without cognitive impairments; and have plans to return to an appointment with a medical oncologist. Breast, colorectal, and lung cancer were chosen as they are among the most common cancers diagnosed in British Columbia and Canada [31]. Recruitment and informed consent were undertaken by a medical oncologist. Consented patients were given the HRQoL and socio-demographic questionnaires at a subsequent outpatient attendance at the Vancouver Cancer Clinic. Data collection occurred between June 2008 and December 2009.

Two options were available to the patients when completing the study. The first option was that the questionnaires be completed face-to-face with a trained research assistant at the patient’s outpatient visit. Alternatively, the patients could take the questionnaires home and return the completed questionnaires in the post in a provided pre-paid envelope. For both options, researchers were available to answer questions if needed. The order of the HRQoL questionnaires was randomized for each participant. The study protocol was approved by the Research Ethics Board of the British Columbia Cancer Agency.

### Health-related quality of life instruments

The FACT-G, EQ-5D and SF-6D were used to elicit information pertaining to HRQoL. Patients also completed a brief socio-demographic questionnaire to obtain information regarding age, sex, marital status, qualification, and ethnicity. Clinical data were obtained from the medical records of patients.

The fourth version of FACT-G consists of 27 Likert-type questions covering four domains: physical well-being (PWB, 7 items), social/family well-being (SWB, 7 items), emotional well-being (EWB, 6 items), and functional well-being (FWB, 7 items) [11]. Summary scores can be calculated for each of these four domains, alongside a single overall score for the instrument.

The EQ-5D consists of a general health descriptive system based on five dimensions and a 100-point visual analogue scale (VAS) [32]. The dimensions cover mobility, self care, usual activities, pain/discomfort, and anxiety/depression and are characterized by three levels (i.e., no problems, some problems and extreme problems). The instrument can be used to describe 243 possible health states, which are assigned utilities based on country-specific algorithms. The most widely used utility algorithm was based on a time trade-off (TTO) survey of 2997 UK respondents [13]. Recently, Shaw et al. developed a utility algorithm based on a TTO survey of 4048 US residents [33], which in lieu of a Canadian algorithm, was used to calculate EQ-5D utilities for this study.

The SF-6D was constructed from a sample of 11 items selected from the Medical Outcomes Study Short Form 36 and has been valued by a representative sample of the UK general population using the standard gamble (SG) valuation technique [34]. The SF-6D is based on a six-dimensional health state classifications that assesses physical functioning, role limitations due to physical health problems, bodily pain, general health, vitality, social functioning, role limitations due to emotional problems, and mental health [35]. Each dimension of the SF-6D has four to six levels and can be used to describe 18,000 health states. In the absence of a Canadian utility algorithm, the UK algorithm was used to calculate the SF-6D preference weights for this study.

### Statistical analyses

The study population was randomly sub-divided into two samples: development and cross-validation. The developmental dataset was used to construct the mapping function, while the latter sample validated the developed algorithm. Demographic and clinical characteristics of the patients assigned to the development and cross-validation samples were compared using chi-squared and t-tests for categorical and continuous variables, respectively. Then Spearman correlation coefficients were calculated to assess the linear relationship between the FACT-G and the two preference-based instruments. We also tested the skewness of the EQ-5D and SF-6D utility scores.

**Figure 1:**
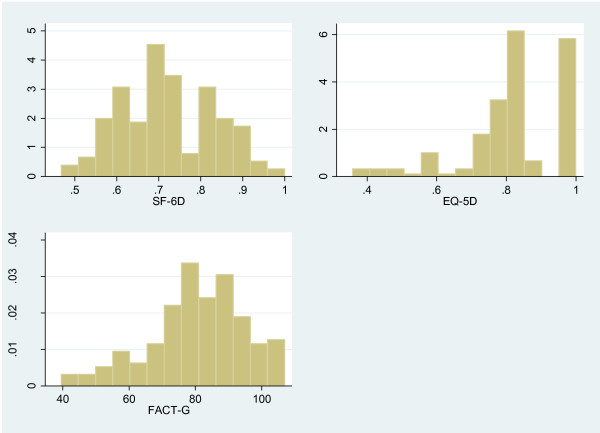
**Distribution of mean SF-6D, EQ-5D and FACT-G scores.**

Ordinary least squares (OLS), generalized linear model (GLM), censored least absolute deviation (CLAD), and the random effects model have been used to derived utilities from preference-based instruments [15,16]. While most previous mapping studies used the OLS model [11,18-41], this approach may not be appropriate when the preference-based scores are highly skewed. (EQ-5D responses are positively skewed for this current study; this is shown in Figure 1). The presence of ceiling effects can lead to inconsistent estimates of the coefficients of independent variables. In the absence of appropriate remedial measures such as suitable transformations, the use of OLS in the presence of heteroscedasticity and non-normality may also be problematic [15].

In this study, we started from the OLS (due to its prevalent use) and calculated robust standard errors that produced consistent estimates in the presence of heteroscedasticity. We extended this to the GLM, which relaxes the assumption of the OLS, to assess whether this approach produced more accurate predictions than the OLS. Finally, we used the CLAD model to account for the ceiling effect. This approach calculates appropriate estimates of the standard error using bootstrapping techniques [42]. Added advantages of the CLAD estimator are its robustness to heteroscedasticity and its consistency. It is also asymptotically normal for a wide class of error distributions and can be used to model data with skewed distributions [42,43]. CLAD is a form of median regression that minimizes the sum of absolute residuals, and as such is not as sensitive to deviations from normality and homoscedasticity [42].

The three regression approaches (i.e., OLS, GLM, and CLAD) were ran for each of the models described below. The approach was to start with the simplest model and then work through more complicated specifications.

### Model 1

The EQ-5D and SF-6D utility indices were each regressed on the FACT-G overall score. The overall score of the FACT-G was rescaled onto a 0-100 scale to enable easier interpretation of the results.

### Model 2

The EQ-5D and SF-6D utility indices were each regressed on the FACT-G domain scores, which were rescaled onto a 0-100 scale.

### Model 3

Demographic (i.e., age and sex) and clinical (i.e., stage of disease) characteristics were introduced to assess whether these variables improved the predictions of the preference-based utilities. Interaction effects (i.e., PWB x FWB, PWB x EWB, age squared, and age x sex) were also tested to examine for any non-linear relationships. However, the interaction effects did not improve the model fit and were not presented. The effect of disease stage and cancer type was explored by including dummy variables in the regression models; the results from this model are discussed in greater detail.

The performances of the models were assessed in terms of how well the responses to the FACT-G predicted the EQ-5D and SF-6D utilities. The adjusted R^2^ describes how well the model explains the dataset it was estimated on; the higher the R^2^, the better the model explains the dataset. The mean absolute error (MAE) and root mean squared error (RMSE) examine the difference between the observed and predicted values, as well as provide an indication of the size of the prediction errors; the lower the values, the better the model is performing [44].

The distributions of the observed and predicted utility scores were evaluated using a Wilcoxon matched-pairs signed-ranks test. The performance of the preferred regression models were compared in terms of their means, standard deviations (SDs), and the 10^th^, 50^th^, and 90^th^ percentiles of the observed and predicted values across two frequently used measures of disease severity: cancer stage (ranging from 1-4) and the Eastern Cooperative Oncology Group (ECOG) performance status (ranging from 0 to 3). For all statistical analyses, STATA version 11.1 was used [45].

## Results

HRQoL information was obtained from 367 patients with breast (n = 140, 38%), colorectal (n = 113, 31%), and lung (n = 114, 31%) cancer. Most of the questionnaires were completed face-to-face (n = 352, 96%). The average age of participants was 58.7 years (SD 11.5 years); 67% were women (Table 1). The patients reported their ethnic compositions as British/Irish (n = 164, 46%), Chinese (n = 53, 15%), or Other (n = 143, 40%). A good representation of disease severity, in terms of cancer stage and ECOG score, was observed in the study sample. There was no statistically significant differences in the demographic and clinical characteristics of the patients in the development (n = 184) and cross-validation samples (n = 183). The majority of patients answered all the items on the HRQoL instruments. The EQ-5D utility indices were negatively skewed, while the SF-6D utilities were mildly skewed; a ceiling effect was observed for the EQ-5D responses (23.4%) (Table 2). The FACT-G score was highly correlated with the EQ-5D (rho = 0.649) and the SF-6D (rho = 0.714) (Table 3).

**Table 1:** **Socio**-**demographic and clinical characteristics of the patients**

	**All (n = 367) count (%)**	**Development (n = 184) count (%)**	**Validation (n = 183) count (%)**
Female	245 (67%)	68%	69%
Mean age (sd)	58.7 (± 11.5)	58.6 (12.5)	58.6 (11.6)
Tumor site			
Breast	140 (38%)	36	35
Lung	114 (31%)	31	33
Colorectal	113 (31%)	33	33
Marital status			
Married or living with partner	251 (70%)	69	71
Single	40 (11%)	11	12
Divorced or widowed	68 (19%)	20	18
Education			
Primary school completed	27 (8%)	10	6
Secondary school completed	108 (30%)	29	27
College or university	211 (59%)	56	65
Other	11 (3%)	4	3
Employment status			
Full time	104 (29%)	30	28
Part time	46 (13%)	12	14
Working at home	10 (3%)	2	2
Retired	133 (37%)	39	37
Unable to work	68 (19%)	17	18
Ethnicity			
British/Irish	164 (46%)	42	45
Chinese	53 (15%)	15	16
Other	143 (40%)	44	38
Disease stage			
Stage 1	39 (11%)	14	11
Stage 2	54 (15%)	17	13
Stage 3	87 (24%)	21	25
Stage 4	178 (50%)	48	51
ECOG			
0	123 (35)	36	34
1	177 (50)	50	51
2	39 (11)	12	10
3	12 (3)	3	3

**Table 2:** Descriptive statistics for instruments used in this study

**Statistics**	**Preference-based measures**		**Cancer-specific instrument**
	**EQ-5D**	**SF-6D**	**FACT-G**
N	363	364	365
Mean	0.82	0.71	78.87
Standard deviation	0.14	0.11	15.47
Median	0.83	0.70	81.00
Minimum	0.11	0.44	36.00
Maximum	1.00	1.00	107
Flooring (%)	0.28	0.27	0.55
Ceiling (%)	23.4	1.37	0.60

**Table 3:** **Spearman correlations between the preference**-**based measures and the FACT**-**G domains**

	**FACT-G**				
	**Global**	**PWB**	**FWB**	**EWB**	**SWB**
**EQ**-**5D**	0.649*	0.631*	0.599*	0.429*	0.222*
**SF**-**6D**	0.714*	0.753*	0.678*	0.386*	0.205*

*p < 0.05.

*PWB*: physical well-being; *FWB*: functional well-being; *EWB*: emotional well-being; *SWB*: social well-being.

### Regression models

#### ***Mapping FACT-G onto EQ-5D***

Model 1: The three regression models (OLS, GLM, and CLAD) showed that the overall FACT-G global score significantly predicted the EQ-5D score (OLS-adjusted R^2^ = 0.331, RMSE = 0.099, MAE = 0.078) (Table 4A, Model 1 for OLS, GLM and CLAD).

**Table 4:** **Regression of the EQ**-**5D and SF**-**6D utility indices upon FACT**-**G**

	**OLS model**			**GLM model**			**CLAD model**		
	**1**	**2**	3*	**1**	**2**	3*	**1**	**2**	3*
**EQ**-**5D**									
FACT-G	0.006^§^			0.008^§^			0.005^§^		
PWB		0.009^§^	0.010^§^		0.012^§^	0.013^§^		0.006^§^	0.012^§^
FWB		0.008^§^	0.006^‡^		0.010^§^	0.007^‡^		0.007^§^	0.005^§^
EWB		0.005^†^	0.006^†^		0.006^†^	0.008^†^		0.005^‡^	0.002^§^
Colorectal^1^			0.018			0.019			0.012
Lung			-0.022			-0.023			0.019
Stage-2^2^			-0.018			-0.021			-0.030
Stage-3			0.019			0.025			0.016
Stage-4			0.026			0.034			-0.009
Constant	0.345	0.391	0.267	-0.798	-0.746	-0.867	0.380	0.481	0.281
Adjusted R^2^	0.331	0.396	0.469						
LL	125.736	136.033	144.088	125.924	136.068	143.054			
RMSE	0.120	0.113	0.104	0.120	0.113	0.111	0.122	0.116	0.095
MAE	0.090	0.086	0.077	0.089	0.085	0.077	0.924	0.086	0.078
N	180	180	162	180	180	162	180	180	164
**SF**-**6D**									
FACT-G	0.005^§^			0.008^§^			0.005^§^		
PWB		0.011^§^	0.011^§^		0.016^§^	0.0159^§^		0.009^§^	0.012^§^
FWB		0.007^§^	0.007^§^		0.011^§^	0.0096^§^		0.008^§^	0.007^§^
EWB		0.001	0.002		0.001	0.003		0.005^‡^	-0.004^§^
Colorectal^1^			0.006			0.002			-0.027
Lung			-0.009			-0.016			-0.050
Stage-2^2^			-0.015			-0.023			-0.031
Stage-3			0.028			0.039			0.016
Stage-4			-0.006			-0.005			-0.022
Constant	0.278	0.343	0.281	-0.982	-0.895	-0.956	0.331	0.282	0.438
Adj_R^2	0.508	0.628	0.651						
LL	207.344	233.806	222.229	209.645	237.754	224.711			
RMSE	0.077	0.066	0.062	0.076	0.065	0.061	0.077	0.069	0.076
MAE	0.062	0.056	0.051	0.061	0.054	0.050	0.062	0.056	0.057
N	182	182	164	182	182	164	182	181	166

*Model 3 -adjusted for age and sex Reference group – ^1^Breast Cancer; ^2^Cancer Stage 1; ^†^p < 0.05, ^‡^p < 0.01, ^§^p < 0.001; *LL* = Log-likelihood; *MAE* = Mean Absolute Error; *RMSE* = Root Mean Square Error. *FACT*-*G*: Functional Assessment of Cancer Therapy; *PWB*: Physical well-being; *FWB*: Functional well-being; *EWB*: Social well-being; *EWB*: Emotional well-being.

Model 2: The PWB, FWB, and EWB domains of the FACT-G significantly predicted the EQ-5D utility indices using the three regression models. Coefficients for the PWB and FWB were highly significant (p < 0.001) compared to the EWB (p < 0.05) (Table 4A, OLS Model 2).

Model 3: After adjusting for the demographic and clinical characteristics of the patients, the three sub-scale scores remained significant predictors of the EQ-5D index score and improved the data fit as explained by higher adjusted R^2^ = 0.469, and lower RMSE = 0.104. In terms of accuracy of prediction, the CLAD model (RMSE = 0.095) performed better compared to the OLS (RMSE = 0.104) and GLM (RMSE = 0.111) (Table 4-A, CLAD Model 3). Adjusting for the type of cancer and disease stage improved the prediction of the model as demonstrated by higher R^2^ (Table 4, OLS Model-3).

#### ***Mapping FACT-G onto SF-6D***

Model 1: A positive and significant correlation was detected between the overall FACT-G global score and the SF-6D utility score using the OLS (adjusted R^2^ = 0.508, RMSE = 0.077, MAE = 0.051). Similar result was found when we used the GLM and CLAD models (Table 4, Model 1).

*Model 2*: The PWB and FWB domains of the FACT-G significantly predicted the SF-6D utility indices (PWB, β = 0.011, p < 0.001; FWB, β = 0.007, p < 0.001) (Table 4, OLS Model 2). The relatively weak correlation between EWB and the SF-6D utility index could be that the emotional aspect is already captured by the other two domains. A significant increase in the amount of variations explained by the sub-scale scores as compared to the overall FACT-G score was observed (increase in the proportion of the total variance explained from adjusted R^2^ = 0.508 to 0.628). Results from the GLM model and the CLAD do not vary much from the OLS model (Table 4, GLM Model; CLAD Model).

Model 3: This model has a higher adjusted R^2^ (0.651) when compared to OLS Models 1 and 2 (Table 4-B). Adjusting for patient characteristics (age and sex) and clinical characteristics (stage of disease in terms of cancer stage) improved the model as demonstrated by a higher adjusted R^2^ and lower RMSE (0.062). Similar results were observed for the GLM and CLAD models. In terms of accuracy of prediction as measured in RMSE, the GLM model (0.061) performed better compared to the CLAD (0.071) and the OLS (0.062) models (Table 4, GLM Model 3). We did not find statistically significant differences in the SF-6D utility index by disease stage and cancer type.

### Comparison of observed and predicted utilities

**Figure 2:**
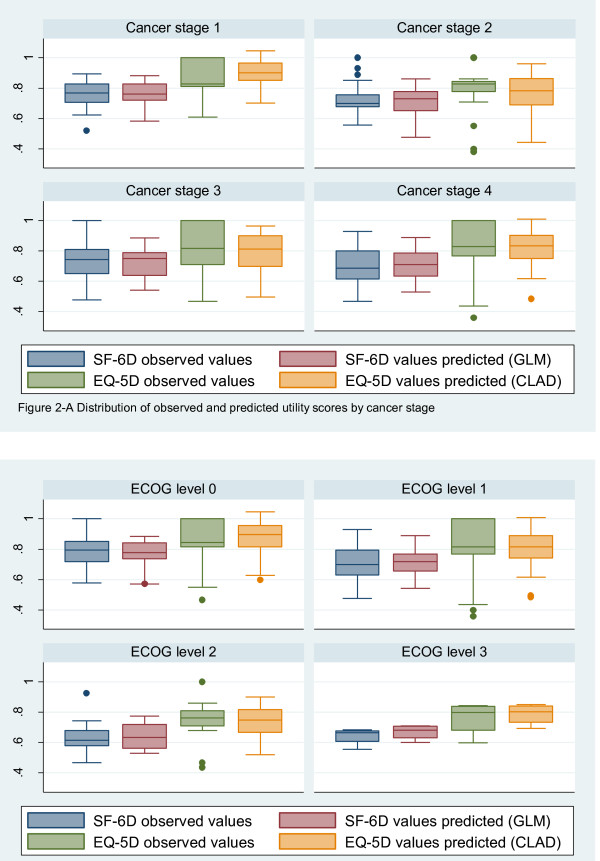
**Distribution of observed and predicted utility scores. (A)** - by cancer stage and **(B)** – by ECOG.

When comparing predictions using the three different regression methods, the CLAD model better predicted the utility scores more closely to the observed values when compared to the predictions obtained using the OLS and GLM models (Table 5). The SF-6D utilities based on GLM followed the observed values more closely. The Wilcoxon matched-pairs signed-ranks test showed no statistically significant differences between the distribution of the observed utility indices and the values predicted by the OLS, GLM, and CLAD models (all p > 0.05). All three models tended to over predict the lower (10^th^ percentile) utility scores and under predict the high (upper 90^th^ percentile) scores (Table 2). Accuracy of predictions across the disease stage measures used in this study (i.e., cancer stage and ECOG performance status) is presented graphically (Figures 2-A and 2-B).

**Table 5:** **Descriptive summary of utility indices derived from observed SF**-**6D**/**EQ**-**5D and OLS**/**GLM**/**CLAD regression models**

	**Mean**	**SD**	**Minimum**	**Percentiles**			**Maximum**
				**10**^%^	**50**%	**90**^%^	
EQ-5D							
Observed	0.823	0.149	0.358	0.768	0.827	1.000	1.000
OLS	0.831	0.103	0.557	0.75	0.84	0.918	0.951
GLM	0.832	0.102	0.583	0.755	0.836	0.921	0.956
CLAD	0.828	0.106	0.511	0.761	0.831	0.905	0.972
SF-6D							
Observed	0.720	0.111	0.467	0.630	0.702	0.799	0.887
OLS	0.723	0.091	0.452	0.658	0.734	0.793	0.839
GLM	0.723	0.092	0.476	0.657	0.730	0.792	0.845
CLAD	0.730	0.102	0.422	0.663	0.744	0.810	0.852

## Discussion

Our results demonstrate that the estimation of both EQ-5D and SF-6D utility indices using the FACT-G responses from breast, lung and colorectal cancer patients can be achieved. We had a relatively large sample of patients with different disease severity levels. Our findings suggested that the CLAD and GLM models are best used to predict EQ-5D and SF-6D utilities, respectively.

Comparing predictions of the preference-based scores using FACT-G scales indicated that a better performance was observed for the SF-6D (adjusted R^2^ = 0.651, MAE = 0.052, RMSE = 0.062) as compared to the EQ-5D (adjusted R^2^ = 0.469, MAE = 0.062, RMSE = 0.104); these results are in agreement with previous studies [11,25-39]. Such studies reported that the physical and functional subscales were significant predictors of the utility scores. For both preference-based indices, including patients’ demographic and clinical characteristics improved explanatory power of the models.

Taking the RSME criteria as measures of prediction, the GLM model using FACT-G domain scores plus patient demographic (age, sex) and clinical (cancer stage) characteristics fitted the SF-6D data well. The GLM model was chosen despite non-Gaussian residuals as they produced the best predictions of utility scores on a natural scale. On the other hand, the CLAD model fitted the EQ-5D data well as compared to the OLS and GLM and it was investigated further.

The PWB and FWB sub-scales of the FACT-G were statistically significant predictors for both preference-based instruments. The EWB reached statistical significance only in the EQ-5D model. The SWB score of the FACT-G was not significantly associated with the EQ-5D and SF-6D and therefore, was not included in the regression model. Similar approaches have been used in previous studies and the similar results have been reported [22]. It is likely that the other dimensions can capture most of the information of other dimensions; as a result, additional information would not be needed. It is possible that only a negligible relationship between those sub-scales and HRQoL exists, and other aspects of illness are captured by them.

For both instruments, Model 3 produced more accurate predictions than Models 1 and 2 demonstrating that including patient characteristics improves predictions. There was a strong correlation between predicted and observed utilities for all three models. Utility scores derived from the FACT-G using the GLM approach were highly correlated with the observed scores (rho = 0.831 for the SF-6D).

The purpose of the current study is to examine the differential validity and prediction of the utility scores using cancer-specific instrument from a sample of breast, lung, and colorectal cancer patients. Our results showed that this is feasible. We also explored this further by assessing differences between observed and predicted utilities by disease severity. Our findings demonstrate that there are similar patterns of prediction by cancer stage, though there is a trend of over predicting values for patients with greater disease severity. Future research is needed to discuss utilization of mixed modeling for patients with different disease severities. While we anticipated that constructing a mapping algorithm using patients with three different tumor sites would be more applicable to facilitate future CUAs in general cancer populations, we found that adjusting for the type and stage of cancer increased the prediction of the model demonstrated by higher R^2^.

The results from this current study are comparable to the one other study that mapped FACT-G responses to EQ-5D in a general cancer population [22]. Similar mean values for the EQ-5D and the FACT-G were observed in the current and previous studies (EQ-5D 0.82 versus 0.81; FACT-G 79.0 versus 81.1, respectively). For the current study, we observed a lower ceiling effect for the EQ-5D (23.4% versus 33.3%) and higher floor effect 0.28%. We observed similar goodness-of-fit values for our OLS Model 3 (R^2^ = 0.47) compared to the previous study (R^2^ = 0.45). In terms of accuracy, we reached same conclusion that predictions using CLAD was more accurate for the EQ-5D compared to the OLS; this was further confirmed when we ran the GLM model and found that the CLAD performs better.

We examined accuracy of predictions across the disease stage measures used in this study (cancer stage and the ECOG). Mean value predictions indicated that overall the GLM model performs better for the SF-6D and the CLAD model performs better for the EQ-5D compared to the alternate methods used in this study. All regression models tend to over predict lower observed utility scores and under predict higher scores.

The poor ability of the FACT-G to predict EQ-5D utilities may be influenced by the brevity of the preference-based instrument (i.e., three levels to define the five dimensions). Our previous work revealed that the EQ-5D was less discriminative between different levels of disease severities when compared to the FACT-G and SF-6D [46]. While the FACT-G, EQ-5D, and SF-6D assesses an individual’s HRQoL, the instruments are composed of different dimensions. For example, the FACT-G and SF-6D has a dimension describing vitality (i.e., an important health outcome for cancer patients), whereas, the EQ-5D does not. To adequately map disease-specific instruments to preference-based measures, a certain degree of overlap is required between the two descriptive systems [15]. If important dimensions are not covered in the preference-based measures, the mapping functions may be compromised. Performing the mapping exercise only using similar dimensions – for example, pain – would not permit the calculation of utilities to be used in CUAs.

The scoring functions of the preference-based instruments were derived from responses of the general population. The EQ-5D and SF-6D consist of dimensions related to individuals’ perceptions of their abilities within a social context (e.g., “I am able to perform my usual activities”). These dimensions capture the ability of the patients to adapt to their health state, which may not be a phenomenon that members of the general population are aware of when faced with an impaired health state [47]. The process of adaptation has been reported to enhance the difference between the utilities for health states provided by patients and members of the general population [48]. Lower utilities tend to be reported by the general population because they do not anticipate their ability to adapt when faced with life with an impaired health state, such as cancer [49]; this may be reflected by a lower magnitude of disutility in the population tariffs. While the use of general population tariffs raises concerns as to whether mapping functions can be estimated accurately for the population used in this study, generated results could be compared independently of the disease. This is one of the reasons why generic preference-based measurements of HRQoL are required for economic evaluations.

The ability of the cancer-specific instruments to predict utilities may be influenced by the psychometric properties of the preference-based measures. While responses on the FACT-G were obtained from Canadian cancer patients, the scoring functions of the EQ-5D and SF-6D are based on non-Canadian populations. These tariffs have been demonstrated that they are valid in a Canadian population [50-53]. However, the constructed mapping algorithms from this study may not be transferrable to patients of other countries. While it is anticipated that the use of a different country’s tariff may only have a marginal effect on the resulting mapping function, the utilities generated may not be appropriate for information resource allocation decisions for the country of study. As such, there is a need to explore the ability of the FACT-G to predict individual dimensions of the EQ-5D and SF-6D; the predicted dimensions could be used to calculate an overall utility score. This has considerable advantages because the derived results are not country-specific and may be more applicable for guiding resource allocation decisions, especially for country that do not have country-specific tariffs for the EQ-5D or the SF-6D.

This study demonstrates that it is possible to facilitate economic evaluations for specific health conditions when a preference-based measure has not been administered and when it would be impractical to conduct a valuation survey using methods such as SG or TTO. This mapping approach offers a shortcut for policy-makers and researchers who need utility values for use in economic evaluation but do not have access to information on preference-based measures. The methodology presented in this study may be applied to other disease-specific instruments.

## Abbreviations

FACT-G: Functional assessment of cancer therapy - general; EQ-5D: EuroQol 5D; SF-6D: Short form 36 health survey; OLS: Ordinary least squares; GLM: Generalized linear models; CLAD: Censored least absolute deviations; MAE: Mean absolute error; RMSE: Root mean squared error; CUA: Cost-utility analysis; QALY: Quality-adjusted life years; HRQoL: Health-related quality of life; CADTH: Canadian agency for drugs and technologies in health; ECOG: Eastern cooperative oncology group; PWB: Physical well-being; SWB: Social/family well-being; EWB: Emotional well-being; FWB: Functional well-being; VAS: Visual analogue scale; TTO: Time trade-off; SG: Standard gamble.

## Competing interests

The authors declare that they have no competing interests.

## Authors’ contributions

PT conceived and designed the study, oversaw all stages of data collection and entry, done the analysis, and drafted the manuscript. SP gave feedback on design and analysis. SP, HM, KvH, SC, BM, KG reviewed the manuscript. All authors read and approved the final manuscript.
